# Drug susceptibility testing of slowly growing non-tuberculous mycobacteria using slomyco test-system

**DOI:** 10.1371/journal.pone.0203108

**Published:** 2018-09-17

**Authors:** Vitalii Litvinov, Marina Makarova, Ksenia Galkina, Elena Khachaturiants, Maria Krasnova, Lidia Guntupova, Svetlana Safonova

**Affiliations:** 1 Department of problems of laboratory diagnostics of tuberculosis and pathomorphology, Scientific and Clinical Antituberculosis Center of Moscow Government Health Department, Moscow, Russian Federation; 2 City consultation diagnostic center, Scientific and Clinical Antituberculosis Center of Moscow Government Health Department, Moscow, Russian Federation; Ross University School of Veterinary Medicine, SAINT KITTS AND NEVIS

## Abstract

**Objective:**

The objective of the research was to assess the susceptibility of the slowly growing nontuberculous mycobacteria strains to the antimicrobial drugs used for mycobaterioses treatment using SLOMYCO test system.

**Materials and methods:**

We assessed 363 NTM strains: 177 MAC (161 *M*. *avium*, 16 *M*. *intracellulare*), 112 *M*. *kansasii* and 74 *M*. *xenopi* collected from the respiratory material of the patients were under the treatment or under diagnostic procedures at our Center, affiliates and the diagnostic department in 2010–2016. Drug sucseptibility for NTM was tested using the Sensititre SLOWMYCO system (TREK DIAGNOSTIC Systems Ltd., UK). MICs were established by microdilutions in Mueller-Hinton broth on polystyrene 96-well plates. The statistical analysis was done using the StatGraphics Plus 5.0 software. The data were compared pairwise using Pearson χ^2^ test with Yates correction. 95% confidence interval (CI) were calculated. Statistically significant differences were considered for p <0.05. Log-rank test and Kaplan-Meier curves were used to assess the concentration-dependent surveillance probability.

**Results:**

The statistically significant differences were revealed in sensitivity/resistance isolates of *M*. *avium* and *M*. *intracellulare*: *M*. *avium* strains were resistant to higher concentrations of amikacin, clarithromycin, linezolid and streptomycin (p <0.01); *M*. *intracellulare* strains were resistant to higher concentrations of ethionamide (p <0.05). The isolates of *M*. *avium* were significantly more resistant than *M*. *kansasii* to amikacin, doxycycline, isoniazid, clarithromycin, linezolid, moxifloxacin, rifabutin, rifampicin, streptomycin, trimethoprim/sulfamethoxazole, ciprofloxacin, ethambutol, ethionamide (visible growth of *M*. *avium* were inhibited by higher drug concentrations, p <0.01). The isolates of *M*. *avium* showed significantly higher resistance than *M*. *xenopi* to amikacin, doxycycline, isoniazid, clarithromycin, linezolid, moxifloxacin, rifampicin, streptomycin, trimethoprim/sulfamethoxazole, ciprofloxacin, ethambutol, and ethionamide (visible growth of *M*. *avium* were inhibited by higher drug concentrations, p <0.01). Statistically significant differences in the dynamics of the response to the antibacterial effects of isoniazid, linezolid, moxifloxacin, rifampicin, trimethoprim/sulfamethoxazole, ethambutol, and ethionamide were found for *M*. *intracellulare* and *M*. *xenopi* (complete inhibition of the visible growth of *M*. *intracellulare* required higher drugs concentrations, p <0, 05). Comparison of the Kaplan-Meyer curves revealed statistically significant differences in survialence probability of *M*. *kansasii* and *M*. *xenopi* for amikacin, doxycycline, rifampicin, trimethoprim/sulfamethoxazole, ciprofloxacin, ethambutol, and ethionamide (a higher number of isolates of *M*. *xenopi* were inhibited by low drugs concentrations, p <0.05).

**Conclusions:**

Our data show that *M*. *avium* and *M*. *intracellulare* were more resistant to the majority of the studied drugs than *M*. *kansasii* and *M*. *xenopi*.

## Introduction

More than 150 species of nontuberculous mycobacteria (NTM) are known worldwide, and many of them cause mycobacterioses in humans [1,2]. In some countries NTM diseases are less common than tuberculosis (TB), but in industrial nations they can be more common than TB [2,3]. In Russia mycobacterioses occur less frequently, but they are often diagnosed as TB due to unawareness of health providers. In the last decades the role of NTM diseases has significantly increased due to HIV-infection. The main causative agents of mycobacterioses in Europe and the US are *Mycobacterium avium-intracellulare* complex (MAC), and in the most regions including Moscow *M*. *kansasii* and *M*. *xenopi* are also commonly spread slowly growing mycobacteria (SGM) [4,5,6,7,8].

Treatment for mycobacterioses is more challenging than TB chemotherapy since many NTM are naturally resistant to the majority of TB drugs also used for mycobacterioses treatment, and there is no standardized antimicrobial treatment for NTM infections [1,2,3,6,8,9].

Drug susceptibility (DS) of NTM is an important phenotypic characteristic, which is essential for an appropriate and effective chemotherapy regimen. Various species of NTM have different profiles of DS. With an increased number of patients needing treatment, the role of drug susceptibility testing is again in the spotlight, however, there are limited data are available on the differences in drug susceptibility profiles between the NTM species [3,9,10].

The methods of drug susceptibility testing (DST) is constantly changing. The use of liquid media allows significantly shortening the incubation period, which results in less degradation and adsorption [9,10,11]. However, due to the expensive equipment and reagents, need for special skills, the test, even performed in the automated systems, are not quite effective for wide implementation (including cost-effectiveness considerations).

The broth microdilution method (Middlebrook 7H9 and 7H12) used in the international study for determination of *M*. *tuberculosis* DS, which results were summed up by U. Eriсsson and J. Sherris [12]. This technique was used for drug susceptibility testing (DST) of rapidly- and slowly growing NTM by J. Swenson et al. Later, this method was widely used for DST of NTM (noncommercial variants, several drugs) [9,13,14,15,16,17,18,19].

Presently, three commercial tests (*TREK Diagnostic Systems Ltd*., UK) are used: *MYCOTB* for *M*. *tuberculosis*, *SLOMYCO* for slowly growing NTM, and *RAPMYCO* for rapidly growing NTM. The major advantages of these test-systems are commercial availability, standardization, easy to set up and the ability of quantitative estimation of the degree of susceptibility/resistance of mycobacteria strains to various concentrations of TB drugs. CLSI published the guidelines advise using broth microdilution assay for DSTs of RGM and SGM. The *SLOMYCO* test system was recommended by CLSI for evaluation the susceptibility of SGM to antimicrobial agents. In studies of many authors have been shown the concordance of the results obtained using the broth microdilution *SLOMYCO* test system and other conventional reference methods for most of the antimicrobial agents. These studies indicate that broth microdilution the *SLOMYCO* Sensititre method could provide a potential alternative to other DST methods [13,15,17,18–25].

## Objective

The objective of the research was to study the antimicrobial susceptibility of the main slowly growing NTM strains to the drugs commonly used to treat the mycobacterioses using *SLOMYCO* test system.

## Materials and methods

The trial was approved by the Ethics Committee of Scientific and Clinical Antituberculosis Center of Moscow Government Health Department, Moscow (number 3, 2016) conducted in accordance with the principles of Good Clinical Practice and the World Medical Association (WMA) Declaration of Helsinki adopted by the 18th WMA General Assembly, Helsinki, Finland, 1964 and subsequent amendments.

### Mycobacterial strains

We studied 363 NTM strains: 177 *MAC* (161 *M*. *avium*, 16 *M*. *intracellulare)*, 112 *M*. *kansasii* and 74 *M*. *xenopi*.

### Patients

All mycobacterial strains were collected from the respiratory material of the patients were under the treatment or under diagnostic procedures at our Center, affiliates and the diagnostic department in 2010–2016. Informed consent was given by each patient for microbiology data processing without publishing any personal data.

### Strains selection

In some cases, several cultures were obtained from the same patients. Only one primary culture was selected from the patient for DST.

### Species identification

Cultures were grown in both solid egg Loewenstein-Jensen medium and Middlebrook 7H9 broth in the automated bacteriological BACTEC 960 system. Species identification involved microbiology (cultures and biochemical tests) and molecular genetic (GenoType CM/AS, Hain Lifescience, Germany) methods according to manufacturers' manuals.

The primary differentiation of the isolated culture of acid-fast mycobacteria for its belonging to the *M*. *tuberculosis complex* or to NTM based on the growth intensity, colony morphology, smear microscopy stained by Ziehl-Neelsen, and also by immunochromatographic "BD MGIT TBc ID" (Becton, Dickinson, USA). Identification of the isolated NTM culture prior to the species was performed by GenoType CM/AS test system (HainLifescience, Germany) and biochemical tests (niacin, nitrate reductase, semi-quantitative catalase, urease, arylsulfatase, Tween-80 hydrolysis, determination of thermostable catalase and potassium telluride potency). The results of identification of the NTM species by microbiological and molecular-genetic methods coincided in 100% of cases. If it was impossible to determine the species of NTM within the avium-intracellulare complex by microbiological methods, only molecular-genetic data were used.

### Drug susceptibility testing

DST of NTM was performed using the *SLOMYCO Sensititre* system (*TREK DIAGNOSTIC Systems Ltd*., UK). MICs were established by microdilutions in Mueller-Hinton broth in polystyrene 96-well plates containing lyophilized drugs in doubly increasing concentrations (μg/ml): amikacin (AMI) 1,0–64,0; doxycycline (DOX) 2,0–16,0; isoniazid (INH) 0,25–8,0; clarithromycin (CLA) 0,06–64,0; linezolid (LZD) 1,0–64,0; moxifloxacin (MXF) 0,12–8,0; rifampin (RIF) 0,12–8,0; rifabutin (RFB) 0,25–8,0; streptomycin (S) 0,5–64,0; trimethoprim/sulfamethoxazole (SXT) 0,12/2,38–8,0/152,0; ciprofloxacin (CIP) 0,12–32,0; ethambutol (EMB) 0,5–16,0; ethionamide (ETH) 0,3–20,0. Detailed information can be found in the study protocol dx.doi.org/10.17504/protocols.io.nu5dey6.

MIC values were defined as the lowest concentration of the drug that inhibited the visible growth of the isolates tested. MIC_50_ and MIC_90_ values were defined as the drug concentration at which 50% and 90% of the isolates tested showed no visible growth, respectively. The MIC breakpoints of antibiotics displaying resistance were interpreted by CLSI.

### Statistical analysis

The statistical analysis was done using the StatGraphics Plus 5.0 software. The data were compared pairwise using Pearson χ2 test with Yates correction. 95% confidence interval (CI) were calculated. Statistically significant differences were considered for p <0.05. Log-rank test and Kaplan-Meier curves were used to assess the concentration-dependent surveillance probability from the first contact with the drug *in vitro* until the complete suppression of mycobacterial growth. The analysis was performed using increasing drug concentration in place of the usual time variable.

## Results

Tables 1–3 show the results of determination of slowly growing NTM susceptibility to the drugs. We used the *SLOMYCO* test system to study the species most prevalent in Moscow region.

**Table 1:** MIC_50_ and MIC_90_ of the drugs used against NTM.

Drug	NTM species, MIC_50_, MIC_90_ (μg/ml)							
	*M*. *avium*		*M*. *intracellulare*		*M*. *kansasii*		*M*. *xenopi*	
	MIC_50_	MIC_90_	MIC_50_	MIC_90_	MIC_50_	MIC_90_	MIC_50_	MIC_90_
**Amikacin**	16.0	32.0	8.0	32.0	8.0	32.0	4.0	16.0
**Doxycycline**	16.0	16.0	16.0	16.0	16.0	16.0	8.0	16.0
**Isoniazid**	8.0	8.0	8.0	8.0	1.0	8.0	1.0	8.0
**Clarithromycin**	4.0	16.0	1.0	4.0	0.5	4.0	0.06	16.0
**Linezolid**	32.0	64.0	16.0	32.0	4.0	32.0	4.0	16.0
**Moxifloxacin**	2.0	4.0	2.0	4.0	0.5	4.0	0.5	2.0
**Rifabutin**	0.5	4.0	0.5	2.0	0.25	2.0	0.25	8.0
**Rifampicin**	4.0	8.0	2.0	8.0	0.25	2.0	1.0	4.0
**Streptomycin**	64.0	64.0	32.0	64.0	16.0	64.0	8.0	64.0
**Trimethoprim /Sulfamethoxazole**	4.0/ 76.0	8.0/ 152.0	4.0/ 76.0	8.0/ 152.0	8.0/ 152.0	8.0/ 152.0	2.0/ 38.0	8.0/ 152.0
**Ciprofloxacin**	16.0	16.0	8.0	16.0	4.0	16.0	1.0	8.0
**Ethambutol**	16.0	16.0	8.0	16.0	4.0	16.0	8.0	16.0
**Ethionamide**	20.0	20.0	20.0	20.0	2.5	20.0	5.0	20.0

MIC_50_ and MIC_90_ were defined as drug concentrations completely inhibiting the visible growth of 50% and 90% of the examined isolates respectively

**Table 2:** The number of drug-resistant strains of NTM*. (the number/percentage of drug-resistant strains and 95% confidence interval (CI), %).

Drug	NTM species												
	*M*. *avium*(1)			*M*. *intracellulare*(2)			*M*. *kansasii*(3)			*M*. *xenopi*(4)			p
	abs.	%	CI	abs.	%	CI	abs.	%	CI	abs	%	CI	
**Clarithromycin**	7	4.4	1.8–8.8	0	0.0	0.0–20.6	2	1.8	0.2–6.3	3	4.1	0.8–11.4	1–2 > 0.05 1–3 > 0.05 1–4 > 0.05 2–3 > 0.05 2–4 > 0.05 3–4 > 0.05
**Linezolid**	119	73.9	66.4–80.5	6	37.5	15.2–64.6	16	14.3	8.4–22.2	6	8.1	3.0–16.8	1–2 < 0.01 1–3 < 0.01 1–4 < 0.01 2–3 > 0.05 2–4 < 0.01 3–4 > 0.05
**Moxifloxacin**	40	24.8	18.4–32.3	4	25.0	7.3–52.4	13	11.6	6.3–19.0	5	6.8	2.2–15.1	1–2 < 0.01 1–3 < 0.05 1–4 < 0.01 2–3 > 0.05 2–4 > 0.05 3–4 > 0.05

* CLSI (2011) criteria were used in calculations

**Table 3:** Comparative data on drug susceptibility of slowly growing NTM.

Drugs	NTM species and the “spectrum” of minimum inhibitory concentrations (MIC)									p
	MIC	*M*. *avium (1)*		*M*. *intracellulare (2)*		*M*. *kansasii (3)*		*M*. *xenopi (4)*		
		n	%	n	%	n	%	n	%	
**Amikacin (AMI)**	1.0	1	0.6	2	12.5	2	1.8	15	20.3	**1–2 < 0.01** **1–3 < 0.01** **1–4 < 0.01** 2–3 > 0.05 2–4 > 0.05 **3–4 < 0.01**
	2.0	3	1.9	1	6.3	4	3.6	8	10.8	
	4.0	10	6.2	1	6.3	31	27.7	22	29.7	
	8.0	39	24.2	7	43.8	37	33.0	16	21.6	
	16.0	62	38.5	3	18.8	19	17.0	8	10.8	
	32.0	31	19.3	1	6.3	13	11.6	4	5.4	
	64.0	15	9.3	1	6.3	6	5.4	1	1.4	
**Doxycycline (DOX)**	0.12	0	0.0	0	0.0	0	0.0	3	4.1	1–2 >0.05 **1–3 < 0.01** **1–4 < 0.01** 2–3 > 0.05 **2–4 < 0.01** **3–4 < 0.01**
	0.25	0	0.0	0	0.0	0	0.0	0	0.0	
	0.5	0	0.0	0	0.0	2	1.8	2	2.7	
	1.0	0	0.0	1	6.3	2	1.8	2	2.7	
	2.0	0	0.0	0	0.0	0	0.0	3	4.1	
	4.0	1	0.6	0	0.0	10	8.9	8	10.8	
	8.0	9	5.6	1	6.3	17	15.2	22	29.7	
	16.0	151	93.8	14	87.5	81	72.3	34	45.9	
**Isoniazid (INH)**	0.25	1	0.6	0	0.0	15	13.4	9	12.2	1–2 > 0.05 **1–3 < 0.01** **1–4 < 0.01** **2–3 < 0.01** **2–4 < 0.01** 3–4 > 0.05
	0.5	2	1.2	0	0.0	23	20.5	9	12.2	
	1.0	7	4.3	1	6.3	22	19.6	22	29.7	
	2.0	6	3.7	0	0.0	13	11.6	12	16.2	
	4.0	9	5.6	1	6.3	11	9.8	11	14.9	
	8.0	136	84.5	14	87.5	28	25.0	11	14.9	
**Clarithromycin (CLA)**	0.06	1	0.6	0	0.0	22	19.6	40	54.1	**1–2 < 0.01** **1–3 < 0.01** **1–4 < 0.01** 2–3 > 0.05 2–4 > 0.05 3–4 > 0.05
	0.12	2	1.2	0	0.0	7	6.3	2	2.7	
	0.25	5	3.1	1	6.3	24	21.4	5	6.8	
	0.5	9	5.6	5	31.3	23	20.5	4	5.4	
	1.0	11	6.8	4	25.0	14	12.5	3	4.1	
	2.0	33	20.5	2	12.5	8	7.1	4	5.4	
	4.0	43	26.7	3	18.8	3	2.7	4	5.4	
	8.0	35	21.7	1	6.3	7	6.3	2	2.7	
	16.0	15	9.3	0	0.0	2	1.8	7	9.5	
	32.0	2	1.2	0	0.0	1	0.9	1	1.4	
	64.0	5	3.1	0	0.0	1	0.9	2	2.7	
**Linezolid (LZD)**	1.0	0	0.0	0	0.0	14	12.5	11	14.9	**1–2 < 0.01** **1–3 < 0.01** **1–4 < 0.01** **2–3 < 0.01** **2–4 < 0.01** 3–4 > 0.05
	2.0	2	1.2	0	0.0	27	24.1	10	13.5	
	4.0	6	3.7	1	6.3	36	32.1	27	36.5	
	8.0	3	1.9	5	31.3	13	11.6	13	17.6	
	16.0	31	19.3	4	25.0	6	5.4	7	9.5	
	32.0	85	52.8	5	31.3	10	8.9	2	2.7	
	64.0	34	21.1	1	6.3	6	5.4	4	5.4	
**Moxifloxacin (MXF)**	0.12	0	0.0	1	6.3	31	27.7	21	28.4	1–2 > 0.05 **1–3 < 0.01** **1–4 < 0.01** **2–3 < 0.05** **2–4 < 0.01** 3–4 > 0.05
	0.25	4	2.5	0	0.0	19	17.0	14	18.9	
	0.5	17	10.6	0	0.0	17	15.2	16	21.6	
	1.0	55	34.2	5	31.3	14	12.5	10	13.5	
	2.0	45	28.0	6	37.5	18	16.1	8	10.8	
	4.0	30	18.6	4	25.0	9	8.0	2	2.7	
	8.0	10	6.2	0	0.0	4	3.6	3	4.1	
**Rifabutin** **(RFB)**	0.25	77	47.8	7	43.8	72	64.3	45	60.8	1–2 > 0.05 **1–3 < 0.01** 1–4 > 0.05 2–3 >0.05 2–4 > 0.05 3–4 > 0.05
	0.5	19	11.8	3	18.8	17	15.2	8	10.8	
	1.0	24	14.9	4	25.0	9	8.0	7	9.5	
	2.0	20	12.4	1	6.3	6	5.4	3	4.1	
	4.0	10	6.2	0	0.0	3	2.7	2	2.7	
	8.0	11	6.8	1	6.3	5	4.5	9	12.2	
**Rifampicin (RIF)**	0.12	1	0.6	1	6.3	14	12.5	8	10.8	1–2 > 0.05 **1–3 < 0.01** **1–4 < 0.01** **2–3 < 0.01** **2–4 < 0.05** **3–4 < 0.05**
	0.25	3	1.9	0	0.0	17	15.2	5	6.8	
	0.5	7	4.3	1	6.3	24	21.4	8	10.8	
	1.0	25	15.5	4	25.0	24	21.4	19	25.7	
	2.0	36	22.4	3	18.8	12	10.7	22	29.7	
	4.0	39	24.2	3	18.8	12	10.7	9	12.2	
	8.0	50	31.1	4	25.0	9	8.0	3	4.1	
**Streptomycin (STR)**	0.5	0	0.0	0	0.0	0	0.0	4	5.4	**1–2 < 0.01** **1–3 < 0.01** **1–4 < 0.01** 2–3 > 0.05 2–4 > 0.05 3–4 > 0.05
	1.0	0	0.0	0	0.0	4	3.6	2	2.7	
	2.0	0	0.0	0	0.0	5	4.5	3	4.1	
	4.0	2	1.2	1	6.3	18	16.1	12	16.2	
	8.0	6	3.7	2	12.5	20	17.9	21	28.4	
	16.0	25	15.5	4	25.0	19	17.0	14	18.9	
	32.0	44	27.3	6	37.5	21	18.8	6	8.1	
	64.0	84	52.2	3	18.8	25	22.3	12	16.2	
**Trimethoprim/Sulfamethoxazole (SXT)**	0.12/ 2.4	2	1.2	0	0.0	1	0.9	8	10.8	1–2 > 0.05 **1–3 < 0.01** **1–4 < 0.01** **2–3 < 0.05** 2–4 > 0.05 **3–4 < 0.01**
	0.25/4.8	1	0.6	0	0.0	0	0.0	5	6.8	
	0.5/9.5	6	3.7	1	6.3	2	1.8	9	12.2	
	1.0/19.0	20	12.4	1	6.3	12	10.7	11	14.9	
	2.0/38.0	35	21.7	4	25.0	4	3.6	10	13.5	
	4.0/76.0	33	20.5	4	25.0	15	13.4	12	16.2	
	8.0/152.0	64	39.8	6	37.5	78	69.6	19	25.7	
**Ciprofloxacin (CIP)**	0.12	1	0.6	0	0.0	0	0.0	2	2.7	1–2 > 0.05 **1–3 < 0.01** **1–4 < 0.01** 2–3 > 0.05 **2–4 < 0.01** **3–4 < 0.01**
	0.25	1	0.6	0	0.0	0	0.0	1	1.4	
	0.5	1	0.6	0	0.0	5	4.5	6	8.1	
	1.0	6	3.7	1	6.3	11	9.8	31	41.9	
	2.0	13	8.1	1	6.3	18	16.1	16	21.6	
	4.0	17	10.6	3	18.8	28	25.0	7	9.5	
	8.0	37	23.0	4	25.0	19	17.0	5	6.8	
	16.0	85	52.8	7	43.8	31	27.7	6	8.1	
**Ethambutol (EMB)**	0.5	1	0.6	0	0.0	3	2.7	3	4.1	1–2 > 0.05 **1–3 < 0.01** **1–4 < 0.05** **2–3 < 0.05** 2–4 > 0.05 **3–4 < 0.01**
	1.0	1	0.6	0	0.0	1	0.9	1	1.4	
	2.0	7	4.3	1	6.3	17	15.2	2	2.7	
	4.0	12	7.5	6	37.5	49	43.8	9	12.2	
	8.0	52	32.3	1	6.3	17	15.2	29	39.2	
	16.0	88	54.7	8	50.0	25	22.3	30	40.5	
**Ethionamide (ETH)**	0.3	0	0.0	0	0.0	14	12.5	3	4.1	**1–2 < 0.05** **1–3 < 0.01** **1–4 < 0.01** **2–3 < 0.01** **2–4 < 0.01** **3–4 < 0.01**
	0.6	1	0.6	0	0.0	15	13.4	3	4.1	
	1.2	1	0.6	0	0.0	21	18.8	4	5.4	
	2.5	7	4.3	0	0.0	15	13.4	13	17.6	
	5.0	26	16.1	1	6.3	12	10.7	17	23.0	
	10.0	17	10.6	0	0.0	9	8.0	11	14.9	
	20.0	109	67.7	15	93.8	26	23.2	23	31.1	

The analysis of the values of MIC_50_ and MIC_90_ for *M*. *avium*, *M*. *intracellulare*, *M*. *kansasii* and *M*. *xenopi* isolates showed a number of differences along with similar results Table 1. Thus, MIC_50_ of amikacin, clarithromycin, linezolid, rifampicin, streptomycin, ciprofloxacin and ethambutol were higher for *M*.*avium* than for other studied NTM and MIC_50_ of isoniazid, moxifloxacin, rifabutin and ethionamide were higher for *M*. *avium* and *M*. *intracellulare* than for *M*. *kansasii* and *M*. *xenopi*, that pointed to a greater degree of resistance of MAC strains to these drugs.

The lowest values of MIC_90_ of amikacin, linezolid, moxifloxacin, rifampicin, and ciprofloxacin were established for *M*. *xenopi*, that demonstrated greater susceptibility of these mycobacterial species.

According to the CLSI criteria, we established the number of strains resistant to three drugs (clarithromycin, linezolid, and moxifloxacin) (Table 2).

There are some important aspects in the CLSI guidelines (2011) to be considered:

the breakpoints for MAC are given for only three drugs (clarithromycin, linezolid, and moxifloxacin) for susceptible, “intermediate,” and resistant strains.

the breakpoints for *M*. *kansasii* are given for more than three drugs (but any comparison is impossible due to the lack of the similar data for MAC).

no data are provided for *M*. *xenopi*, that is why we used the breakpoints established for *M*. *kansasii*.

The data presented in Table 2 showed that clarithromycin had a high inhibitory activity against all the studied species of NTM, and almost all strains were susceptible to it. There were no significant differences in drug susceptibility of *M*.*avium*, *M*.*intracellulare*, *M*. *kansasii* and *M*.*xenopi* isolates to clarithromycin (p> 0.05).

The ratio of the resistant NTM to linezolid and moxifloxacin significantly differ between the strains: the higher number of *M*. *avium* strains were resistant to both drugs than the number of *M*. *intracellulare*, *M*. *kansasii* and *M*.*xenopi* strains (p <0.01).

Significant differences were also shown for the ratio of the linezolid-resistant strains of *M*. *intracellulare* and *M*.*xenopi*. There were significantly higher linezolid-resistant strains of *M*. *intracellulare* (p <0.01).

Thus, the data above show that the most strains of *M*.*avium*, *M*.*intracellulare*, *M*. *kansasii* and *M*.*xenopi* were susceptibile to clarithromycin. The significant differences were revealed in susceptibility to linezolid and moxifloxacin. These results should be taken into account while choosing appropriate chemotherapy regimen for the patients with mycobacteriosis caused by these NTM species.

**Fig 1:**
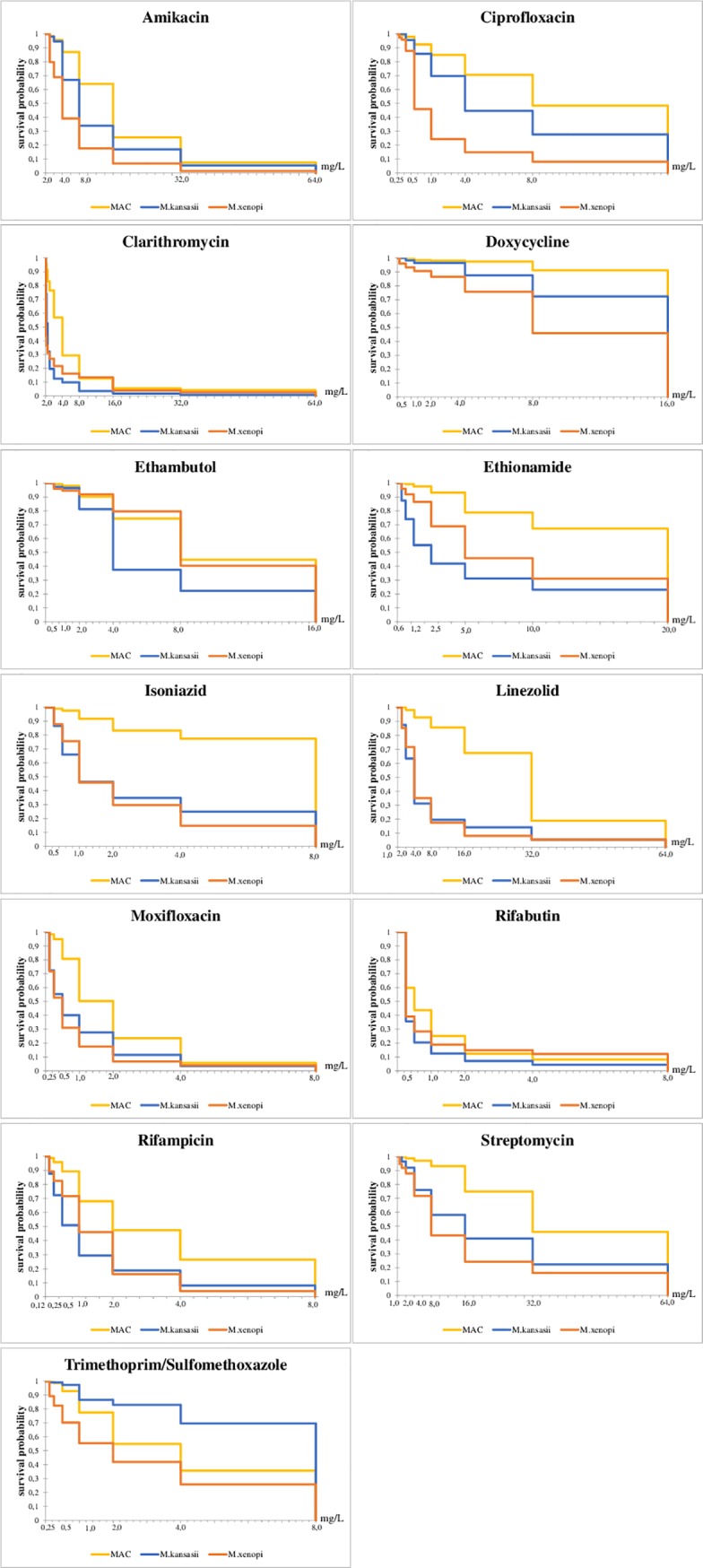
The comparison of Kaplan-Meier survival curves for MICs of drugs used against NTM.

The analysis of Kaplan-Meier survival curves constructed for clinical isolates of *M*.*avium*, *M*.*intracellulare*, *M*. *kansasii*, and *M*. *xenopi*, showed the following Table 3, Fig 1.

The *M*.*avium* and *M*. *intracellulare* isolates had similar sensitivity/resistance profile to the set of the studied drugs, significant differences were found for amikacin, clarithromycin, linezolid and streptomycin (*M*. *avium* strains were resistant to higher drug concentrations, p <0.01) and ethionamide (*M*. *intracellulare* strains were resistant to higher drug concentrations, p <0.05).

The survival of *M*.*avium* and *M*. *kansasii* was significantly different under amikacin, doxycycline, isoniazid, clarithromycin, linezolid, moxifloxacin, rifabutin, rifampicin, streptomycin, trimethoprim/sulfamethoxazole, ciprofloxacin, ethambutol, ethionamide (for complete suppression of *M*.*avium* higher concentrations of these drugs were required, p <0.01).

Comparison of survival curves of *M*.*avium* and *M*. *xenopi* showed significant differences in susceptibility to amikacin, doxycycline, isoniazid, clarithromycin, linezolid, moxifloxacin, rifampicin, streptomycin, trimethoprim/sulfamethoxazole, ciprofloxacin, ethambutol, and ethionamide (higher concentrations of these drugs were required to completely suppress *M*.*avium* growth, p <0.01).

Significant differences in the dynamics of the response to the effects of isoniazid, linezolid, moxifloxacin, rifampicin, trimethoprim/sulfamethoxazole, ethambutol, and ethionamide were found for *M*. *intracellulare* and *M*. *xenopi* (were required higher concentrations of these drugs for complete suppression of *M*. *intracellulare* growth, p <0, 05).

Comparison of the survival curves of *M*. *kansasii* and *M*. *xenopi* revealed significant differences for amikacin, doxycycline, rifampicin, trimethoprim/sulfamethoxazole, ciprofloxacin, ethambutol, and ethionamide (low concentrations of these drugs inhibited the growth of a higher number of strains of *M*. *xenopi*, p <0.05).

In general, the results of this study indicate that all the studied species of NTM showed the same sensitivity or resistance only to certain drugs. Strains of *M*.*avium* and *M*. *intracellulare* were more often resistant than *M*. *kansasii*, *M*. *xenopi* to most drugs.

## Discussion

DSTs of NTM have proven to be clinically useful in select settings, but most of their role remains unknown. For many drugs, relationships between in vitro activity and in vivo outcomes of treatment have not been studied. Due to the differences between even individual NTM strains, these organisms require individualized treatment that must be selected on the basis of results obtained from in vitro drug susceptibility tests [3,10,15,17].

There are many publications devoted to MIC of the drugs described in our research (*MAC*, *M*. *kansasii*, *M*. *xenopi*). To determine MIC the authors predominantly used the proportions in agar and BACTEC 460, 960 with liquid media [1,8,10,15], and the broth microdilution method [9,10,13,17,26,27], including *SLOMYCO Sensititre* [18,25].

Our data show that the information obtained by the other authors about MIC and numbers (%) of resistant strains was either consistent with our data or differed from them, more or less.

The range of MIC and values of MIC_50_ and MIC_90_ of the most drugs (amikacin, rifabutin, rifampicin, streptomycin, ciprofloxacin, ethambutol, ethionamide) for *Mycobacterium avium-intracellulare* complex and MIC_50_ and MIC_90_ of the most drugs (amikacin, linezolid, moxifloxacin, rifabutin, streptomycin, ciprofloxacin, ethambutol, ethionamide) for *M*. *kansasii* were practically consistent with the previously published data [9,10,13,14,18,25,27,28].

For example, in a study carried out in China [27], the MIC_90_ of clarithromycin for *M*. *intracellulare* was 2.0 μg/ml. The MIC_50_ of clarithromycin for *M*. *kansasii* was 2.0 μg/ml, MIC_90_ was 4.0 μg/ml [28]. According to H. Duan et al. [27] MIC_90_ of linezolid for *M*. *intracellulare* was 64.0 μg/ml; according to B. Brown-Elliott et al. [5] MIC_50_ for MAC was 32.0 μg/ml, MIC_90_−64.0 μg/ml, and according to B. Brown-Elliott et al. [9], MIC_50_ and MIC_90_ for *M*. *kansasii* were 2.0 and > 2.0 μg/ml, respectively. According to T. Wu et al. [26], MIC_50_ of moxifloxacin against *M*. *kansasii* was 2.0 μg/ml, MIC_90_ was > 8.0 μg/ml.

The results obtained in the system *SLOMYCO* [18,25] slightly differ (to one or the other direction) from our data. Thus, the study carried out in Sweden [25] showed the range of MIC of clarithromycin for MAC strains were 0.06–128.0 μg/ml with a MIC_50_ of 2.0 μg/ml; MIC of linezolid in the range 1.0–128.0 μg/ml with a MIC_50_ of 32.0 μg/ml, MIC of moxifloxacin in the range 0.25–16.0 μg/ml with a MIC_50_ of 2.0 μg/ml.

Information on the number (%) of resistant *MAC* cultures with a relatively high probability is available only for clarithromycin, linezolid and moxifloxacin (by comparison with CLSI breakpoints, 2011). At the same time, it can be stated that almost complete coincidence has been noted only for linezolid. In the present study more resistant cultures of MAC have been found [8,10,13, 14, 18,25,28].

In the studies conducted in Brazil [29], Taiwan [28], and in Iran [29] it was established that all cultures of *M*. *kansasii* were susceptible to clarithromycin; by P. Heidarich et al.[29], all cultures of *M*. *kansasii* were susceptible to linezolid and moxifloxacin, and, according to T. Wu et al. [26], 59.5% of *M*. *kansasii* strains were susceptible, and 40.5% of them were resistant to moxifloxacin. F. Alcaide et al. [30] found that all the studied strains of *M*. *kansasii* were susceptible to clarithromycin, linezolid, and moxifloxacin. According to H. Duan et al. [27], 93.4% of *M*. *intracellulare* strains were susceptible, and 6.6% of *M*. *intracellulare* strains were resistant to clarithromycin; 32.9% of them were susceptible to linezolid, 22.4% had an intermediate sensitivity/resistance, and 44.7% of strains of *M*. *intracellulare* were resistant.

We should bring into focus that it is critical to perform such an analysis in each region/country on a regular basis to gain a “platform” for mycobacterioses treatment considering drug susceptibility/resistance profile and its trend typical for the area.

Despite the limitations associated with the difficulties of visual interpretation of the results (less reproducibility) and the lack of interpretation categories (breakpoints) for some drugs and types of NTM, the use of the SLOMYCO test system allows testing of clinical isolates of SGM with several drugs at several concentrations without much labor and the use of expensive equipment.

## Supporting information

S1 Table M. kansasii database. (XLSX)

S2 Table M.xenopi database. (XLSX)

S3 Table MAC database. (XLSX)
